# Genome-wide identification and analyses of the rice calmodulin and related potential calcium sensor proteins

**DOI:** 10.1186/1471-2229-7-4

**Published:** 2007-01-30

**Authors:** Bongkoj Boonburapong, Teerapong Buaboocha

**Affiliations:** 1 Department of Biochemistry, Faculty of Science, Chulalongkorn University, Payathai Road, Patumwan, Bangkok 10330, Thailand

## Abstract

**Background:**

A wide range of stimuli evoke rapid and transient increases in [Ca^2+^]_cyt _in plant cells which are transmitted by protein sensors that contain EF-hand motifs. Here, a group of *Oryza sativa *L. genes encoding calmodulin (CaM) and CaM-like (CML) proteins that do not possess functional domains other than the Ca^2+^-binding EF-hand motifs was analyzed.

**Results:**

By functional analyses and BLAST searches of the TIGR rice database, a maximum number of 243 proteins that possibly have EF-hand motifs were identified in the rice genome. Using a neighbor-joining tree based on amino acid sequence similarity, five loci were defined as *Cam *genes and thirty two additional *CML *genes were identified. Extensive analyses of the gene structures, the chromosome locations, the EF-hand motif organization, expression characteristics including analysis by RT-PCR and a comparative analysis of *Cam *and *CML *genes in rice and Arabidopsis are presented.

**Conclusion:**

Although many proteins have unknown functions, the complexity of this gene family indicates the importance of Ca^2+^-signals in regulating cellular responses to stimuli and this family of proteins likely plays a critical role as their transducers.

## Background

Ca^2+ ^is an essential second messenger in all eukaryotic cells in triggering physiological changes in response to external stimuli. Due to the activities of Ca^2+^-ATPases and Ca^2+^-channels on the cellular membrane, rapid and transient changes of its cytosolic concentrations are possible. In plant cells, a wide range of stimuli trigger cytosolic [Ca^2+^] increases of different magnitude and specialized character [1,2], which are typically transmitted by protein sensors that preferably bind Ca^2+^. Ca^2+ ^binding results in conformation changes that modulate their activity or their ability to interact with other proteins. For the majority of Ca^2+^-binding proteins, the Ca^2+^-binding sites are composed of a characteristic helix-loop-helix motif called an EF hand. Each loop, including the end of the second flanking helix, provides seven ligands for binding Ca^2+ ^with a pentagonal bipyramid geometry. Ca^2+^-binding ligands are within the region designated as +X*+Y*+Z*-Y*-X**-Z, in which * represents an intervening residue. Three ligands for Ca^2+ ^coordination are provided by carboxylate oxygens from residues 1 (+X), 3 (+Y) and 5 (+Z), one from a carbonyl oxygen from residue 7 (-Y), and two from carboxylate oxygens in residue 12 (-Z), which is a highly conserved glutamate (E). The seventh ligand is provided either by a carboxylate side chain from residue 9 (-X) or from a water molecule.

In plants, three major groups of Ca^2+^-binding proteins that have been characterized include calmodulin (CaM), Ca^2+^-dependent protein kinase (CPK), and calcineurin B-like protein (CBL) [3-5]. Recently, Reddy ASN and colleagues have analyzed the complete Arabidopsis genome sequence, identified 250 genes encoding EF-hand-containing proteins and grouped them into 6 classes [6]. CaM, a unique Ca^2+ ^sensor that does not possess functional domains other than the Ca^2+^-binding motifs belongs to group IV along with numerous CaM-related proteins. CaM is a small (148 residues) multifunctional protein that transduces the signal of increased Ca^2+ ^concentration by binding to and altering the activities of a variety of target proteins. The activities of these proteins affect physiological responses to the vast array of specific stimuli received by plant cells [7]. In plants, one striking characteristic is that numerous isoforms of CaM may occur within a single plant species. A large family of genes encoding CaM and closely related proteins from several plants has been identified, however, with the exception of Arabidopsis, families of genes encoding CaM and related proteins have not been extensively conducted in a whole-genome scale. In addition, a very limited number of studies on individual rice CaMs has been published [8-10].

With the completion of the genomic DNA sequencing project in *Oryza sativa *L., all sequences belong to a multigene family such as CaM and related proteins can be identified. Preliminary searching of *Oryza sativa *L. databases revealed numerous genes encoding CaM-like proteins. In Arabidopsis, McCormack and Braam [11] have characterized members of Groups IV and V from the 250 EF-hand encoding genes identified in the Arabidopsis genome. Six loci are defined as *Cam *genes and 50 additional genes are CaM-like (*CML*) genes, encoding proteins composed mostly of EF-hand Ca^2+^-binding motifs. The high complexity of the CaM and related calcium sensors proteins in Arabidopsis suggests their important and diverse roles of Ca^2+ ^signaling. It would be interesting to know how this family of proteins exists in the genome of rice which is considered a model plant for monocot and cereal plants because of its small genome size and chromosomal co-linearity with other major cereal crops. In this study, we identified genes encoding proteins that contain EF-hand motifs and are related to CaM from the rice genome. Analyses of the identified gene and protein sequences including gene structures, chromosomal locations, the EF-hand motif organization and expression characteristics as well as comparison with Arabidopsis *Cam *and *CML *genes were carried out.

## Results and Discussion

### Identification and phylogenetic analysis of EF-hand-containing proteins

To identify EF-hand-containing proteins, firstly, we functionally searched the *Oryza sativa *L. genome at The Institute for Genomic Research (TIGR) [12] for Interpro Database Matches by five different methods including HMMPfam, HMMSmart, BlastProDom, ProfileScan and superfamily as described in the "Methods" section. Secondly, we searched the rice database using the amino acid sequences of rice CaM1 [10] and CBL3 [13] as queries in the programs BLASTp and the protein sequences that were not found by the domain searches were added to the list. In addition, we reviewed literature on reports of EF-hand-containing proteins in rice that have been identified by various methods. All of these protein sequences were again analyzed for EF hands and other domains using InterProScan [14]. InterProScan is a protein domain identifying tool that combines different protein signature recognition methods from the consortium member databases of the Interpro [15]. As a result, domain searches identified 245 proteins but six sequences did not have an EF hand identifiable by InterProScan using default settings, so they were eliminated from further analysis. BLAST searches have found four more EF-hand-containing proteins and literature review has yielded no additional proteins. Totally, a maximum of 243 putative EF-hand-containing proteins in rice have been identified [see Additional file 1]. Nearly half of these proteins contain no other identifiable domains predicted by InterProScan. It should be noted that 24 proteins contain a single EF-hand motif that was identified by only one prediction program and could be false positives.

**Figure 1:**
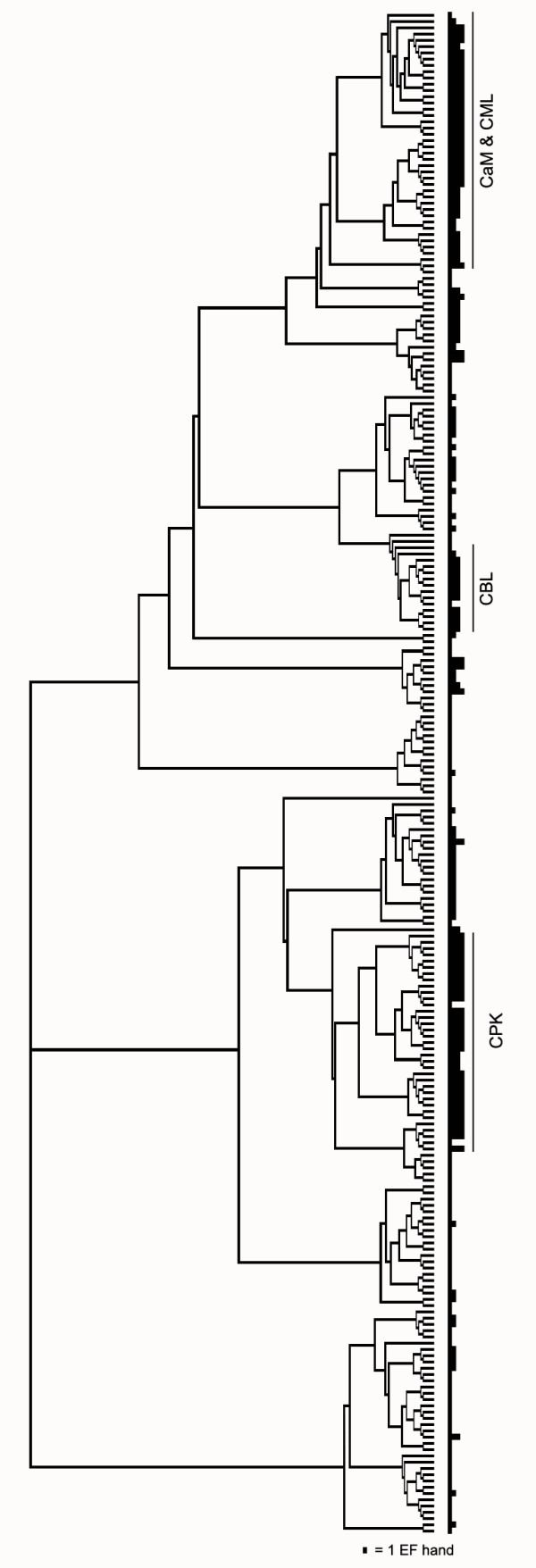
**Phylogenetic tree showing the overall relatedness of EF-hand-containing proteins in rice**. Alignment of full-length protein sequences and phylogenetic analysis were performed as described in the "Methods" section. The numbers of EF hands predicted by InterProScan for each protein are shown as black blocks on the right with their heights proportional to their numbers of motif. With the exception of two proteins, all proteins indicated by the vertical line labelled "CaM & CML" at the right share more than 25% amino acid identity with OsCaM1 and were selected for further analyses. Positions of CBL and CPK members are also shown along the tree to emphasize their separation.

Next, sequences of all the proteins identified by the InterProScan as containing an EF-hand motif were aligned using Clustal X [16] [see Additional file 2]. Tree construction using the neighbor-joining method and bootstrap analysis was performed. Figure 1 shows the tree outline illustrating the numbers of EF hands predicted by InterProScan for each protein on the right without any gene identifiers. As a result, proteins that do not possess functional domains other the Ca^2+^-binding EF-hand motifs were found distributed across the tree but most were concentrated in the top half. Conversely, most proteins in the bottom half contain additional domains that give clues to their functions which include transcription factor, ion channel, DNA- or ATP/GTP-binding protein, mitochondrial carrier protein, protein phosphatase and protein kinase. Two known major groups of EF-hand-containing proteins: calcineurin B-like (CBL) [13] and Ca^2+^-dependent protein kinase (CPK) proteins [17] are separately grouped as shown in Figure 1. We observed that most of the proteins containing four EF-hand motifs are either in the CPK group or located at the top of the tree surrounding the typical CaM proteins. With the exception of two, all proteins indicated by "CaM & CML" share at least 25% amino acid identity with OsCaM1 and were selected for further analyses. This list should contain rice proteins that are related to CaM or has functions based on Ca^2+^-binding mode similar to CaM. Existence of these genes and their deduced amino acid sequences were confirmed using another annotation database, the Rice Annotation Project Database (RAP-DB) [18].

### Rice CaM proteins

**Figure 2:**
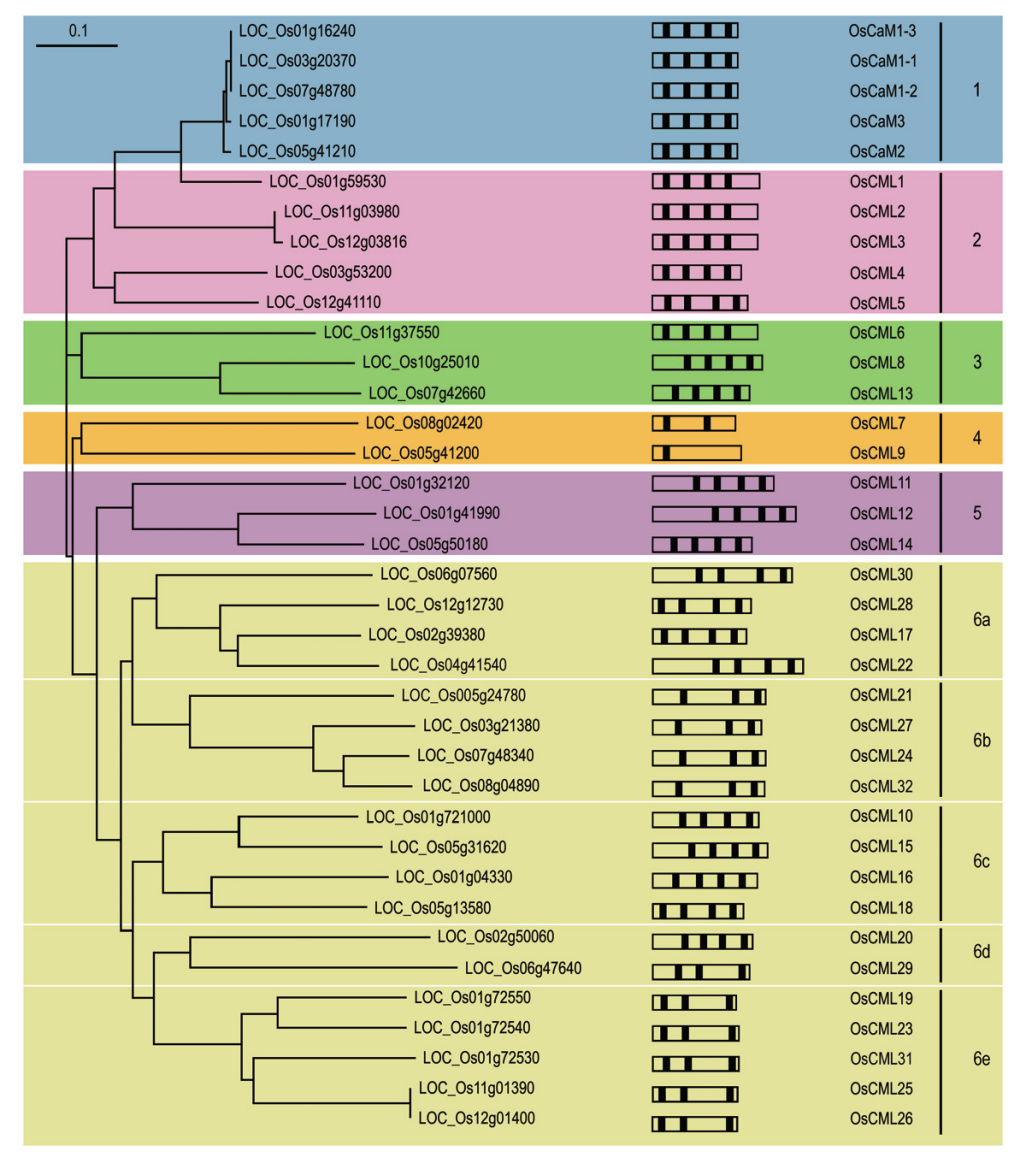
**Neighbor-joining tree based on amino acid similarities among OsCaM and OsCML proteins**. Tree construction using the neighbor-joining method and bootstrap analysis was performed with ClustalX. The TIGR gene identifier numbers are shown and the resulting groupings of CaM and CaM-like proteins designated as 1–6 are indicated on the right. Schematic diagrams of the OsCaM and OsCML open reading frames show their EF hand motif distribution.

The full-length amino acid sequences of the selected proteins were subjected to phylogenetic analysis. Tree construction using the neighbor-joining method and bootstrap analysis performed with ClustalX [see Additional file 3] generated a consensus tree which is depicted in Figure 2. This analysis led us to separate these proteins into six groups: 1–6. What defines a "true" CaM and distinguishes it from a CaM-like protein that serves a distinct role *in vivo *is still an open question. Different experimental approaches including biochemical and genetic analyses have been taken to address this question [19]. In this study by phylogenetic analysis based on amino acid sequence similarity, five proteins in group 1 that have the highest degrees of amino acid sequence identity (≥ 97%) to known typical CaMs from other plant species were identified. Because of these high degrees of amino acid identity, we classified them as "true" CaMs that probably function as typical CaMs. They were named OsCaM1-1, OsCaM1-2, OsCaM1-3, OsCaM2 and OsCaM3. Their characteristics are summarized in Table 1.

**Figure 3:**
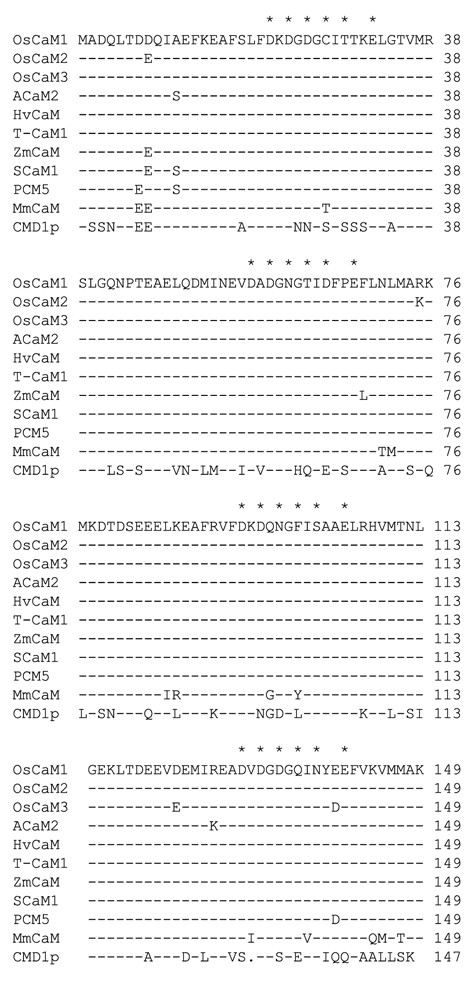
**OsCaM protein sequence similarity with CaM from other species**. Comparison of the deduced amino acid sequences of OsCaM1, 2, and 3 with those of other plants, *Mus Musculus *CaM (MmCaM), and *Saccharomyces cerevisiae *CaM (CMD1p). The sequences are compared with OsCaM1 as a standard; identical residues in other sequences are indicated by a dash (-), and a gap introduced for alignment purposes is indicated by a dot (.). Residues serving as Ca^2+^-binding ligands are marked with asterisks (*).

*OsCam1-1*; *OsCam1-2 *and *OsCam1-3 *encode identical proteins, whereas *OsCam2 *and *OsCam3 *encode a protein of only two amino acid differences and their sequences share 98.7% identity with those of OsCaM1 proteins. Multiple sequence alignment of the OsCaM amino acid sequences with those of typical CaMs from other species shown in Figure 3 indicates their high degree of sequence conservation. It should be mentioned that OsCaM1 amino acid sequences are identical to those of typical CaMs from barley (*H. vulgare*) and wheat (*T. aestivum*) reflecting the close relationships among monocot cereal plants. On average, OsCaM amino acid sequences share about 99%, 90% and 60% identity with those from plants, vertebrates and yeast, respectively. Hydrophobic residues contributing to hydrophobic interaction in the mechanism of CaM-target protein complex formation which are critical to CaM function are highly conserved. All of the conserved eight methionine (M) and nine phenylalanine (F) residues among plant CaMs are present in all OsCaMs. Conservation of these residues is maintained between plant and vertebrate CaMs, with the exception of the methionine residues at position 145–146 in plants CaMs, which are displaced one residue compared with the vertebrate proteins. Due to its considerable conformational flexibility [20] and being weakly polarized, methionine residues which are estimated to contribute nearly half of the accessible surface area of the hydrophobic patches of CaM allow it to interact with target proteins in a sequence-independent manner [21]. Sequence conservation related to functionality of plant CaMs also includes lysine (K) at position 116 which is assumed to be trimethylated. All OsCaM proteins possess a lysine residue at this position. Lysine 116 trimethylation is believed to be a posttranslational modification that helps regulate CaM activity. EF-hand motifs will be discussed later in the "number and structure of EF hand" subsection.

The presence of multiple CaM isoforms is a defining characteristic of CaMs in plants. Even though the explanation of gene redundancy still cannot be ruled out, accumulating evidence suggests that each of the *Cam *genes may have distinct and significant functions. Previous reports have shown that highly conserved CaM isoforms actually modulate target proteins differently [22]. Induced expression of some but not all of the multiple CaM isoforms in a plant tissue in response to certain stimuli has been reported [10,23] thus, competition among CaM isoforms for target proteins may be found. It is fascinating that the *OsCam1-1*, *OsCam1-2*, and *OsCam1-3 *genes encode identical proteins. How these protein sequences have been maintained with the natural selection pressure throughout evolution has no clear answer yet but it is likely that each of these genes has physiological significance.

### Rice CaM-like (CML) proteins

The remaining proteins from the phylogenetic analysis in Figure 2 were named CaM-like or CML according to the classification by McCormack and Braam [11]. Like CaM, these proteins are composed entirely of EF hands with no other identifiable functional domains. A summary of their characteristics is shown in Table 1. They were named according to their percentages of amino acid identity with OsCaM1 which were calculated by dividing the number of identical residues by the total number of residues that had been aligned to emphasize the identical amino acids. These proteins are small proteins consisting of 145 to 250 amino acid residues and sharing amino acid identity between 30.2% to 84.6% with OsCaM1. All the CML proteins in group 2 share more than 60% of amino acid sequence identity with OsCaM1. The CML proteins in groups 3, 4, and 5 have identities with OsCaM1 that average 48.2%, 46.9%, and 43.8%, respectively. By the bootstrapped phylogenetic tree based on amino acid sequence similarity of these proteins, group 6 CML proteins were separated into five subgroups: 6a-6e. These proteins share identities no more than 40.7% with OsCaM1 that average at 35.6% with the exception of OsCML10 (45.6%). All members of groups 6b and 6e contain three EF-hand motifs though with different configurations.

Some important CaM functional features were found existing only in a few CaM-like proteins. The characteristic cysteine (C) at residue 7(-Y) of the first EF hand, a hallmark of higher plant CaM sequences is absent in all CaM-like proteins with the exception of three highly conserved CML proteins, which are OsCML4, OsCML5 and OsCML6. Based on multiple sequence alignment, OsCML4, OsCML5, OsCML7 OsCML10, OsCML17, OsCML18, and OsCML28 are the only CaM-like proteins that contain lysine at a position equivalent to the Lys116 of CaMs. These features may be indicators of proteins that serve similar *in vivo *functions with those of CaMs. OsCML4 and OsCML5 are the only CaM-like proteins that possess both of these signature characteristics. However, another important determinant of CaM function, which is a high percentage of methionine (M) residues, has been found in most of the OsCML proteins. The average percentage of M residues among OsCMLs is 4.6% compared with 6.0% in OsCaMs. Considering the usually low percentage found in other proteins, the Met-rich feature in CMLs is likely an indication of their relatedness to CaMs and possibly similar mechanisms of action i.e. exposure of hydrophobic residues caused by conformational changes upon Ca^2+ ^binding. Nonetheless, some newly attained characteristics specific to CMLs probably allow them to fine-tune their Ca^2+^-regulated activity to more specialized functions.

Of these proteins, three OsCMLs contain an extended C-terminal basic domain and a CAAX (C is cysteine, A is aliphatic, and X is a variety of amino acids) motif, a putative prenylation site (CVIL in OsCML1 and CTIL in OsCML2 and 3). OsCML1, also known as OsCaM61 was identified as a novel CaM-like protein by Xiao and colleagues [8]. The CML protein was reported to be membrane-associated when it is prenylated and localized in the nucleus when it is unprenylated [9]. A similar protein called CaM53 previously found in the petunia also contains an extended C-terminal basic domain and a CAAX motif which are required for efficient prenylation [24]. Similar subcellular localization of CaM53 depending on its prenylation state was reported. To locate another possible modification, all proteins were analyzed by the computer program, Myristoylator [25]. As a result, OsCML20 was predicted to contain a potential myristoylation sequence. No other potential myristoylated glycines either terminal or internal were found among the rest of the OsCML proteins. In addition, to determine the possible localization of the OsCML proteins, their sequences were analyzed by targetP [26]. OsCML30 was predicted to contain an endoplasmic reticulum signal sequence and OsCML21 was predicted to be an organellar protein. For OsCaMs and other OsCMLs, no targeting sequence was present, thus, they are probably cytosolic or nuclear proteins

### Number and structure of EF hand

**Figure 4:**
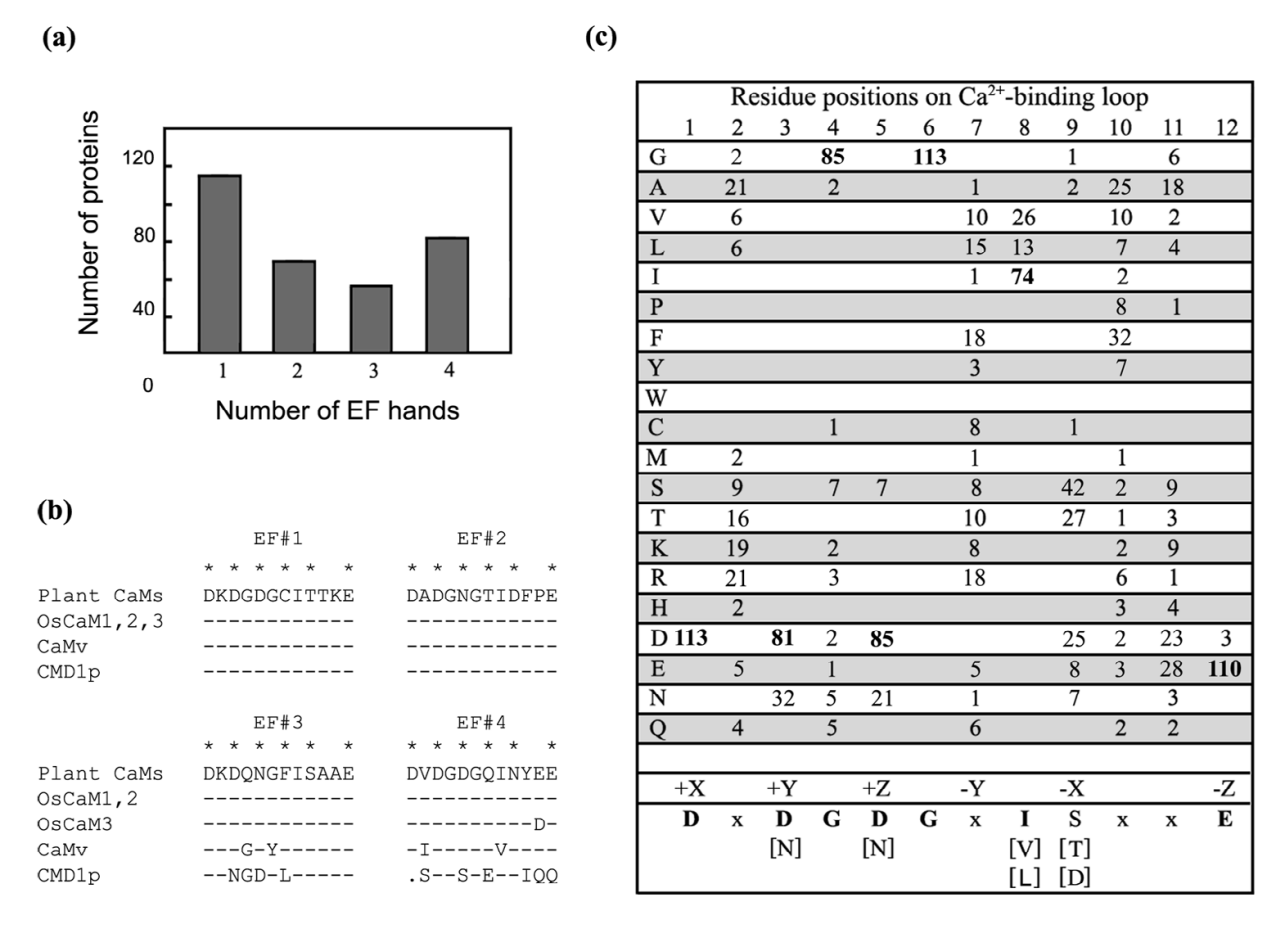
**Characteristics of EF hands in rice proteins**. (a) Number of EF-hand-containing proteins containing 1, 2, 3 or 4 EF hands. (b) Residues in the EF hands #1-4 of OsCaMs compared with those of typical plant CaMs, vertebrate CaM (CaMv) and *Saccharomyces cerevisiae *CaM (CMD1p) using a consensus sequence of plant CaMs as a standard; identical residues in other sequences are indicated by a dash (-), and a gap introduced for alignment purposes is indicated by a dot (.). (c) Residues in Ca^2+^-binding loops in 32 OsCML proteins shown as the frequency at which an amino acid (shown at the left) is found in each position (shown at the top). The amino acids most frequently found are indicated by bold letters and shown below as a consensus sequence along with the positions of residues serving as Ca^2+^-binding ligands indicated in Cartesian coordinates. Bracketed residues are alternative residues frequently found in each position and "x" is a variety of amino acids. Residues serving as Ca^2+^-binding ligands are marked with asterisks (*).

The number of EF hands in the rice EF-hand-containing proteins varied from 1 to 4. A summary of the number of proteins having 1, 2, 3, or 4 EF hands is shown in Figure 4a. It turned out that among the 243 proteins identified, almost all proteins that contain 4 EF hands were included in our study or are CPK proteins. All five OsCaM proteins have two pairs of EF hands with characteristic residues commonly found in plant CaMs. Consensus sequence of the Ca^2+^-binding site in the EF hands of plant CaMs compared with OsCaM1, OsCaM2, OsCaM3, vertebrate CaM, and CMD1p from yeast is shown in Figure 4b. Ca^2+^-coordinating residues among OsCaMs are invariable with those of the plant CaM consensus sequence. Other residues in the Ca^2+^-binding loop are also conserved with only the exception of aspartate (D) at residue 11 of the fourth EF hand in OsCaM3. Among the twenty EF-hand motifs of OsCaMs, residues 1(+X) and 3(+Y) are exclusively filled with aspartate (D); residues 5(+Z) are aspartate (D) and asparagine (N); and residues 12(-Z) are glutamate (E) which is invariably found in this position of most Ca^2+^-binding EF hand motifs. This residue may rotate to give bidentate or monodentate metal ion chelation. Glutamate provides two coordination sites, favoring Ca^2+ ^over Mg^2+ ^coordination [27]. Residues 7(-Y) are usually varied; and residues 9(-X) are aspartate (D), asparagine (N), threonine (T), and serine (S) which are all normally found among plant CaMs.

Schematic diagrams of each protein sequence with the predicted EF hands represented by closed boxes are shown in Figure 2. Among all the identified OsCaM and OsCML proteins, about three fourths of the EF hands that exist in pairs (59 pairs) are interrupted by 24 amino acids. The rest are positioned at a similar distance relative to each other which is between 25–29 amino acids with the exception of two pairs that are less than 24 amino acids apart. Most OsCML proteins have either two pairs or at least one pair of identifiable EF hands except OsCML9 which has a single EF hand and OsCML7 which appears to have two separate EF hands. OsCML7 and OsCML9 are interesting because of their high amino acid identities with OsCaM1 (47.7% and 46.1%) but they possess only 2 and 1 EF hands; and have relatively low methionine (M) content (2.8% and 3.2%) compared with other OsCML proteins, respectively. In addition, 10 OsCML proteins with one pair of identifiable EF hands have an extra EF hand that does not pair with any other motif. Pairing of EF-hand motifs in the CaM molecule helps increase its affinity for Ca^2+^, therefore an unpaired EF hand in these proteins may bind Ca^2+ ^with a lower affinity, or may be non-functional.

Ligands for Ca^2+ ^coordination in the EF-hand motifs of OsCML proteins are highly conserved. One hundred and thirteen Ca^2+^-binding sequences were aligned and the frequency at which amino acids were found is tabulated in Figure 4c. Most residues in the Ca^2+^-binding loops are conserved among OsCML proteins, thus suggesting that most of them are functional EF hands. Similar to OsCaMs, residues 1(+X) are exclusively filled with aspartate (D); and residues 3(+Y) and 5(+Z) are usually aspartate (D) or asparagine (N). Even though they are not coordinating residues, glycine (G) at position 6 is absolutely conserved and hydrophobic residues (I, V, or L) are always found at position 8 in all 133 EF hands in OsCaM and OsCML proteins. Residues 12(-Z) are mostly glutamate (E) with the exceptions of an EF hand in OsCML7, OsCML8, and OsCML13 which have aspartate (D) instead. While OsCML8 and OsCML13 have two pairs of EF-hand motifs, OsCML7 possess two separate EF hands with D at residue 12 in the EF-hand motif at the carboxyl terminus. Cates and colleagues [27], previously reported that mutation of E12 to D reduced the affinity of EF hands for Ca^2+ ^in parvalbumin by 100-fold and raised the affinity for Mg^2+ ^by 10-fold. It is likely that these EF hands bind Mg^2+^rather than Ca^2+ ^but the physiological significance of Mg^2+^-binding CaM-like activity is still not known.

### *Cam *and *CML *gene structures and chromosomal distribution

**Figure 5:**
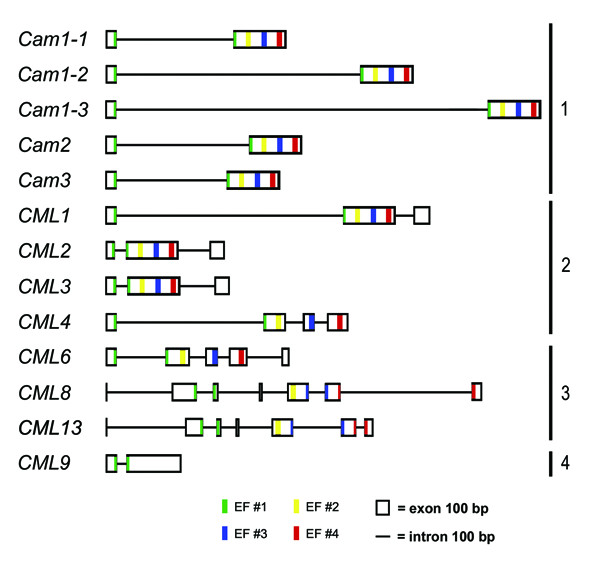
**Schematic representation of the *OsCam *and *OsCML *genes**. Boxes represent exons and lines represent introns. EF-hand motif #1, #2, #3, and #4 are represented by green, yellow, blue and red stripes at their positions, respectively. Groupings of the genes are shown on the right.

The structures of the *OsCam *and *OsCML *genes were mapped by comparing their full length cDNAs with the corresponding genomic DNA sequences. In cases where no full length cDNA was available, partial cDNA and EST sequences were used. Their results were compared and verified with the annotation at the TIGR database. Out of 37 *OsCam *and *OsCML *genes, 13 genes contain intron(s) in their coding regions in which none of these is found in group 5 and 6 members. It should be mentioned that by TIGR annotation *OsCam1-2 *and *OsCML1 *genes were shown to have an alternatively spliced mRNA that encodes a slightly different protein with little supporting evidence so they were eliminated from our list. Schematic diagrams depicting intron-exon structures of the intron-containing genes are shown in Figure 5. All *OsCam *genes contain a single intron which interrupts their coding regions within the codon encoding Gly26, a typical rearrangement of all plant *Cam *genes.

Interestingly, all of the intron-containing *OsCML *genes are also interrupted by an intron at the same location as *OsCam *genes. The conservation of this intron position indicates their close relationships which is consistent with the fact that these genes encode members of the CML proteins groups 1-4, closely-related CaM-like proteins to OsCaMs. *OsCML1*, *OsCML2*, and *OsCML3 *genes contain an additional intron that resides at the codon corresponding to the last residue of genes encoding conventional CaMs. These proteins have an extended C-terminal basic domain and a putative prenylation site. The position of these introns reflects the separation of functional domains within these proteins and suggests that the sequences encoding their carboxyl extensions arose later in the evolution by the fusion of existing *Cam *genes to the additional exons. Similarly, *OsCML8 *and *OsCML13 *which encode group 3 proteins have the same gene structure which is the same intron number (6) and location. The gene duplication event that led to the existence of *OsCML8 *and *OsCML13 *is also supported by the high degree of amino acid identity (60%) between OsCML8 and OsCML13. In these proteins, one of the six introns locates within the sequence encoding the third EF-hand motif, a location comparable to Gly26 of the first EF-hand motif. This intron is probably the remnant of a duplication event that originally gave rise to two EF-hand pairs in these proteins. Interestingly, OsCML8 and OsCML13 are two out of only three OsCMLs that contain aspartate (D) at residues 12(-Z). These observations suggest that the mutation of E12 to D in OsCML8 and OsCML13 probably occurred before the duplication event that led to their existence.

**Figure 6:**
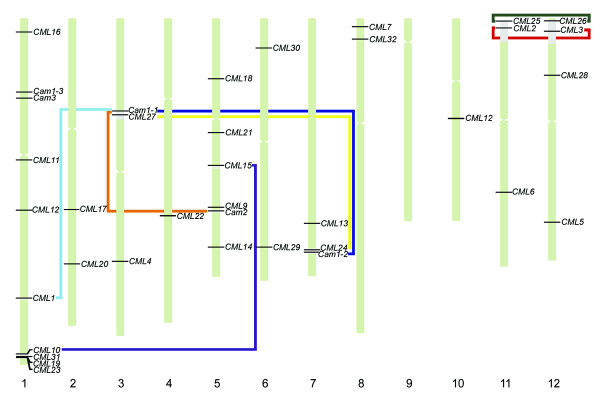
**Chromosomal distribution of the *OsCam *and *OsCML *genes in the rice genome**. The chromosome numbers are shown at the bottom; horizontal lines represent the respective genes; and the centromeric regions appear constricted. Regions of the predicted segmental duplications are indicated by grey sections in the chromosomes and lines connecting the affected loci.

The chromosomal location of each gene was determined from the annotation at the TIGR database. *OsCam *and *OsCML *genes were found distributed across 11 chromosomes of rice as shown in Figure 6 with chromosome 1 having the most numbers (10) of genes. *OsCam1-1 *was mapped in chromosome 3, *OsCam1-2 *in chromosome 7; *OsCam1-3*, and *OsCam3 *in chromosome 1; and *OsCam2 *in chromosome 5. Their nucleotide sequences share between 86–90 % identities which are lower than their amino acid identities (98–100%). Multiple *OsCam *genes encoding nearly identical proteins have been maintained through natural selection suggesting the functional significance of each gene. *OsCam1-1 *and *OsCam1-2 *which encode identical proteins were mapped to the duplicated regions of chromosome 3 and 7, respectively. *OsCam1-1 *and *OsCam2 *were also located within duplicated genome segments of their respective chromosomes. These observations suggest that these pairs of genes are derived from segmental duplication. In addition, there are many pairs/groups of *OsCML *genes which encode proteins that share a high degree of amino acid identity (≥ 60%). OsCML2/OsCML3 (98.9% identical) and OsCML25/OsCML26 (100% identical) are the most closely related pairs. *OsCML2 *and *OsCML3 *encode potential Ca^2+^-binding proteins in group 2 with an absolute conservation of the C-terminal sequences that contain a prenylation site (CTIL). *OsCML2 *and *OsCML25*; and *OsCML3 *and *OsCML26 *were mapped to the recently duplicated regions of chromosomes 11 and 12, respectively. Therefore, *OsCML2*/*OsCML3*; and *OsCML25*/*OsCML26 *may have arisen through the segmental duplication event. Other pairs/groups of closely related CaM-like genes that are likely to be derived from gene duplication events are *OsCML1*/*OsCam1-1*; *OsCML10*/*OsCML15*;*OsCML24*/*OsCML27*; and *OsCML19*/*OsCML23*/*OsCML31*. All members in each pair or group have the same number and positions of EF-hand motifs. The positions of predicted segmental duplication according to the analyses by TIGR are illustrated along with the chromosomal locations of the affected genes in Figure 6. Conversely, *OsCML19*, *OsCML23 *and *OsCML31 *are arranged in tandem orientation on chromosome 1 suggesting that they were derived from tandem duplication. Interestingly, *OsCML27 *is adjacent to *OsCam1-1 *on chromosome 3 and its duplicated gene, *OsCML24*, resides in tandem with *OsCam1-2 *(OsCaM1-1 and OsCaM1-2 are 100% identical). Therefore, a local duplication followed by a segmental duplication possibly occurred.

### Comparative analysis of rice and Arabidopsis *Cam *and *CML *genes

**Figure 7:**
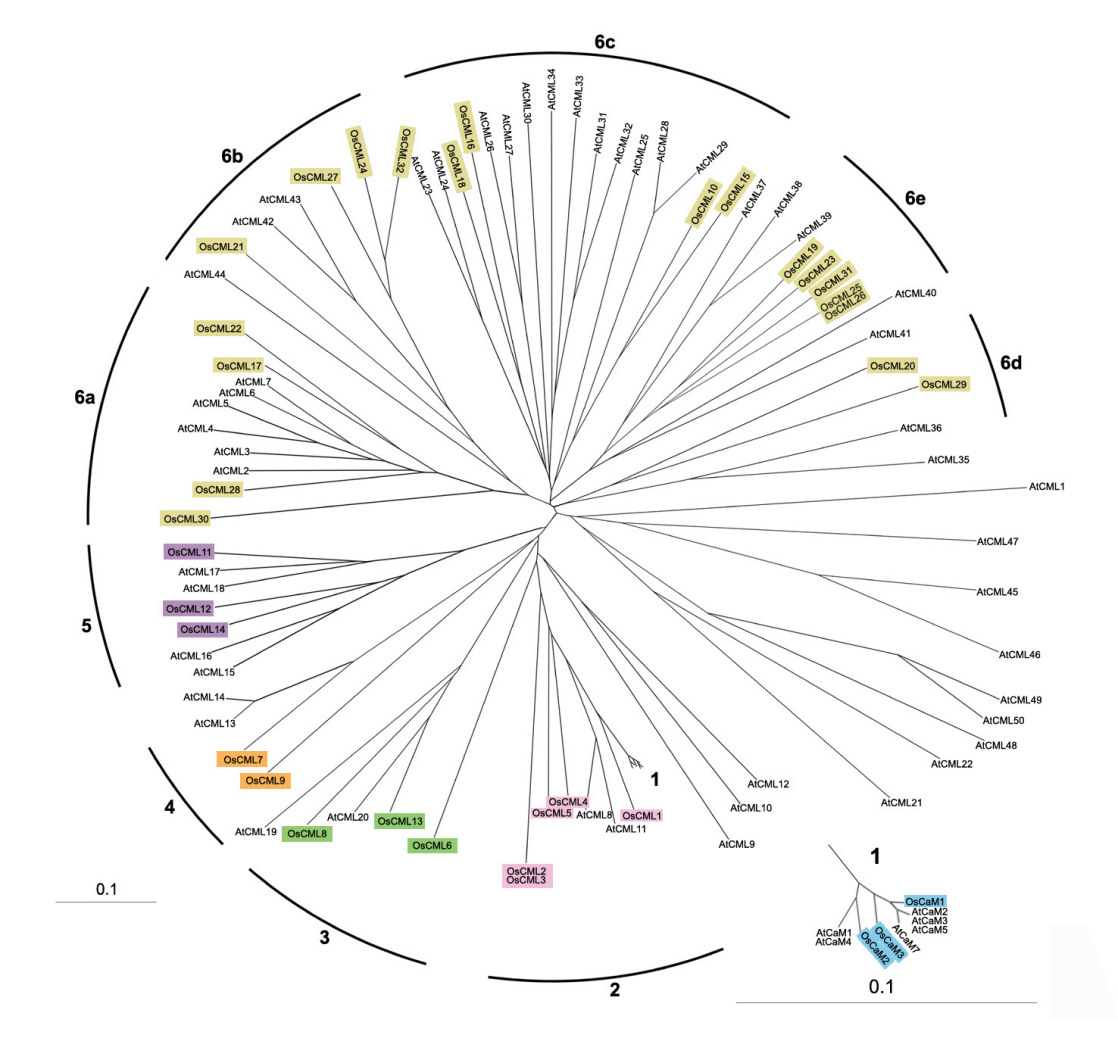
**Phylogenetic relationships among rice and Arabidopsis CaM and CML proteins**. Tree construction using the neighbor-joining method and bootstrap analysis was performed with ClustalX based on the amino acid similarities among the proteins. Rice protein names are highlighted with colours representing each group as used in Figure 2 for clarity and groupings of OsCaM and OsCML proteins are indicated accordingly. OsCaM (group 1) and AtCaM portion of the tree was expanded and shown in the bottom right corner.

The full-length amino acid sequences of rice CaMs and CMLs and Arabidopsis CaMs and CMLs were subjected to phylogenetic analysis. Tree construction using the neighbor-joining method and bootstrap analysis was performed with ClustalX [see Additional file 4]. In Arabidopsis by the neighbor joining tree based on amino acid similarities, McCormack and Braam [11] divided CaMs and CMLs into 9 groups. We found that several rice CaMs and CMLs shared high levels of similarity with Arabidopsis CaMs and CMLs and displayed relationships among the family members similar to those previously reported in Arabidopsis as shown in Figure 7. All of OsCaM proteins in Arabidopsis and rice are highly conserved (sharing 96.6%–99.3% identity). Interestingly, both Arabidopsis and rice have three *OsCam *genes that encode identical proteins (*ACaM2*, *3*, *5 *and *OsCam1-1*, *1-2*, *1-3*). Rice CMLs groups 2, 3, 4, and 5 proteins were closely related to Arabidopsis CMLs group 2, 5, 3, and 4, respectively. The more divergent rice CMLs groups 6a to 6e are also distributed among members of Arabidopsis CML groups 6, 7, 8, 6, and 9, respectively. Apparently, groups 1 from both species are embedded in groups 2. These resulted from the arbitrary separation of groups 1 (CaMs) even though group 2 members share very high degrees of identity (at least 50%) with group 1 proteins. Because what defines a "true" CaM and distinguishes it from a CaM-like protein that serves a distinct role *in vivo *is still unknown, therefore at the moment, only members that share extremely high degrees of identity (>97%) were grouped together to emphasize that they were considered and are possible "true" CaMs.

Based on amino acid sequence alignments (data not shown), many of OsCMLs have putative homologues in Arabidopsis. In group 2, OsCML4 which shares a high level of identity with AtCML8 and AtCML11 has the same number (3) and locations of introns except that AtCML11 lacks the first intron. Similarly, *AtCML19 *and *AtCML20 *which share a high level of identity with their homologues (*OsCML8 *and *OsCML13 *in group 3) have a similar gene structure which is the conservation of five out of the six introns present in their rice counterparts. Interestingly, AtCML19/20 and OsCML8/13 proteins have aspartate (D) at residues 12(-Z) in one of their EF hands, though not on the same hand. AtCML13 and AtCML14, which were thought to have a common progenitor, have very high level of identity (74.3% and 70.9%) with group 4 OsCML7 and all have the mutation of E12 to D in an EF hand corresponding to the third EF hand position. However, OsCML7 has lost an EF hand corresponding to the second position while a second E12 to D mutation was found in AtCML13 and AtCML14. Therefore, similar to AtCML13 and AtCML14, OsCML7 has only one EF hand with canonical amino acids which may result in an impaired ability to bind Ca^2+^. In OsCML group 5, OsCML11 is closely similar to AtCML17 and AtCML18 and, interestingly all have a relatively low percentage of methionine (M) compared with other CML proteins that share similar levels of identity with CaMs. OsCML11 has only 1.4% methionine content which suggests that its mode of action upon Ca^2+ ^binding is probably different from the hydrophobic surface exposure upon conformational changes of CaM.

Previous reports identified 250 EF-hand-containing proteins from the Arabidopsis genome [6]. Seven loci were defined as *Cam *genes and 50 additional genes were *CML *genes [28]. Here, we identified 243 EF-hand-containing proteins, five *Cam *genes and 32 *CML *genes. Fewer members of rice CMLs were identified and several Arabidopsis CMLs did not fall into any group of the rice proteins probably because rice OsCML proteins we included in these analyses had at least 25% identity with typical CaMs compared to 16% in Arabidopsis by McCormack & Braam (2003). We noticed that all of the Arabidopsis proteins that did not fall into any group of the rice proteins shared only 16–30 % identity with typical CaMs. Therefore, both plants appear to have more or less similar numbers of EF-hand-containing and CaM-like proteins. Both also have similar numbers of *CPK *(34 in Arabidopsis and 29 in rice) and *CBL *genes (10 in both Arabidopsis and rice) [13,29]. However, one strikingly different characteristic that we observed is the three OsCML proteins (OsCML1, OsCML2, and OsCML3) that have the carboxyl-terminal CAAX motif for prenylation but none was found in CMLs from Arabidopsis [11]. It would be interesting to know what functions these rice proteins possess and how the prenylation state affects their activity.

### *Cam *and *CML *expression

Because the presence of cDNA or EST clones indicates expression of the corresponding genes, we performed searches against the cDNA/EST rice databases. The searches revealed that majority of the *OsCam *and *OsCML *genes have corresponding cDNA or EST clones. We have identified all the EST clones for each of the *OsCam *and *OsCML *genes. Characteristics of their expression can be inferred according to which libraries the EST clones were derived from. A summary of the numbers of EST clones found in different organs is presented in Table 2. Based on the availability of their EST clones, most *OsCam *and *OsCML *genes are expressed. Some *OsCML *genes are highly expressed in specific organs compared with other genes such as *OsCML13 *and *OsCML18 *in floral tissues. No cDNA or EST clone is available for *OsCML6*, *OsCML19*, *OsCML23*, and *OsCML25*. However, it is not conclusive that these genes do not express relying solely on the absence of their EST clones. Nonetheless, the availability of EST clones for the rest of the *OsCam *and *OsCML *genes indicate that they are expressed and indeed are functional genes.

**Figure 8:**
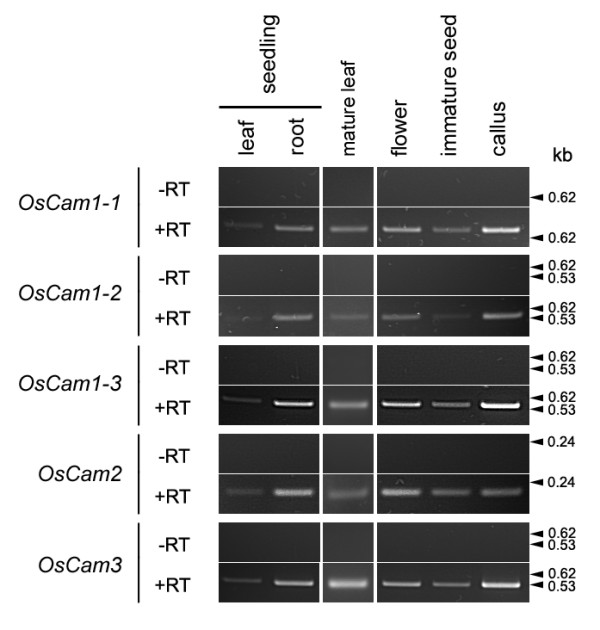
**Expression pattern of the *OsCam *genes**. The total RNA isolated from organs indicated was used in RT-PCR assays either without (-RT) or with (+RT) the addition of M-MLV reverse transcriptase. The cDNAs were amplified by PCR using gene-specific primers as shown in Table 3. The products derived from 250 ng of total RNA were separated in agarose gels and visualized by ethidium bromide staining. The sizes of DNA markers in base pairs are shown on the right.

Because five *OsCam *genes encode only three different proteins, whether or not they have different physiological functions is an interesting question. Here, we experimentally determined whether the expression of each of the *OsCam *genes is restricted to specific organs. Total RNA was isolated from the leaves, roots, flowers, immature seeds and calli of rice plants and used to perform reverse transcription and PCR amplification reactions. Primers selected by computer analysis of the cDNA and EST sequences corresponding to these genes are given in Table 3. A control RT-PCR reaction without adding reverse transcriptase was done in parallel with each experimental reaction to ensure that the product obtained could be attributed to the product of the reverse transcriptase reaction. Figure 8 shows that bands of the expected sizes based on each of the gene sequences (698, 526, 551, 201, and 520 base pairs for *OsCam1-2*, *OsCam1-2*, *OsCam1-3*, *OsCam2*, and *OsCam3*, respectively) were detected in all organs or tissues examined including the leaves and roots of 2-week old seedlings, mature leaves, flowers, immature seeds and calli. No band was detected in the control RT-PCR reactions. It should be noted that the RT-PCR conditions used in this study did not allow quantitative determination, therefore comparison of the expression levels among different organs or different genes can not be made. Nevertheless, it can be concluded that all of *OsCam *genes were expressed in all organs that we examined.

The expression of closely related *Cam *genes in a single organ was not surprising. Several similar occurrences in other plant species have been reported. In tobacco, all 13 *Cam *closely related genes were expressed in almost all organs examined with a few exceptions, notably *NtCam13*, which was exclusively expressed in the root [23]. However, *NtCam13 *encodes a protein of less than 80% identity to typical plant CaMs. Similarly, *ACam1-ACam5 *genes which encode nearly identical proteins were all expressed in the leaves and siliques of Arabidopsis [30,31]. While *Cam *expression is ubiquitous among different cells, protein concentrations may vary in specific cell types. Immunolocalization studies have shown that root cap cells and meristematic zones have increased CaM accumulation [32]. In addition, levels of steady state transcripts of *Cam *genes have been reported to be modulated at different developmental stages [33,34] and in response to external stimuli such as salinity, wind, cold, wounding and pathogen attack [23,35-37]. *OsCam1-1 *was shown to be rapidly and strongly increased in leaves under osmotic stress [10,38]. Modulation of expression in specific organs of a CaM isoform possibly serves its roles in a timely fashion.

## Conclusion

We identified 243 proteins that possibly have EF-hand motifs and 37 CaMs and related potential calcium sensor proteins in the rice genome. The functions of most proteins encoded by these genes are still unknown. Nonetheless, the complexity of CaM protein family likely reflects the importance of Ca^2+ ^signals in regulating cellular responses to various cellular stimuli and this family of proteins potentially plays a critical role. The present results can lead to further studies on each member of this family which will be invaluable in understanding the mechanisms of Ca^2+^-regulated signal transduction pathways in rice.

## Methods

### Database searches and analyses of gene structures and chromosomal distribution

Searches of the rice genome at The Institute of Genomic Research (TIGR) [39] for Interpro Database Matches by five different methods including HMMPfam, HMMSmart, BlastProDom, ProfileScan, and superfamily were carried out. Proteins shown to contain an EF-hand motif or in the family of Ca^2+^-binding proteins which included domains PF00036, SM00054, PD000012, PS50222, and protein family SSP47473, respectively by each method were collected. In addition, BLAST searches (blastp) [40] using the protein sequences of rice CaM1 [GenBank: NP_912914] and CBL3 [GenBank: NP_643248] as query sequences against the rice genome were conducted. Nucleotide and amino acid sequences as well as information regarding each gene of interest were obtained. Gene annotations at the Rice Annotation Project Database (RAP-DB) [41] were also used to confirm the existence and sequences of these genes. Gene structure and locations were determined by a comparison of cDNA and genomic DNA sequences obtained from GenBank and searches of the identified loci at TIGR. Information from EST sequences was used when any discrepancy was found. Gene duplication was determined according to the analysis of chromosomal segmental duplication of the rice genome by TIGR.

### Alignments and tree construction

If necessary, predictions of coding regions were verified using available EST and cDNA sequences. Deduced sequences of proteins identified by InterProScan as containing an EF hand were subjected to phylogenetic analysis. Alignments were performed by the multiple sequence alignment program ClustalX [16] using default settings. Alignments were carried out and protein trees were constructed using the neighbor-joining method [42] with bootstrap analysis by Clustal X (default settings). A comparison of OsCaM proteins with those from other species by multiple sequence alignment was performed by ClustalW. GenBank accession numbers for the sequences used in the alignment are as follows: ACaM2 [GenBank: AAA32763]; HvCaM [GenBank: AAA32938]; T-CaM1 [GenBank: AAC49578]; ZmCaM [GenBank: CAA52602]; SCaM1 [GenBank: AAA34013]; PCM5 [GenBank: AAA85155]; MmCaM [GenBank: NP_033920]; CMD1p [GenBank: AAA34504].

### Amino acid identity and motif analyses of proteins

Deduced amino acid sequences CaM and CaM-like proteins were aligned with one another by Align [43] and the percentage of amino acid identity was calculated by dividing the number of identical amino acids by the total number of amino acid residues of the aligned sequences. All of the protein sequences were analyzed for EF hands and other domains using InterProScan [44]. Positions of the EF hands were located using information from the prediction by InterProScan and by comparing the complete sequences of all proteins with the plant EF-hand consensus sequence. All identified EF hand sequences were aligned with ClustalX and a consensus sequence was generated. To locate sequences for protein modification and targeting, computer programs: Myristoylator [45] and targetP [46] were used.

### Expressed Sequence Tags

ESTs corresponding to *Cam *and *CML *genes were identified by performing BLAST searches of the *Oryza sativa *EST database and by searching UniGene entries corresponding to all genes at GenBank [47]. Expression characteristics of all genes were determined based on the types of library from which ESTs were derived and from literature reviews.

### Analysis by Reverse Transcription Polymerase Chain Reaction (RT-PCR)

*Oryza sativa *L. tissues were ground in liquid nitrogen using chilled mortars and pestles. Total RNA was isolated according to [48] and used in reverse transcription. Reverse transcription was primed by oligo(dT)_15 _primers and PCR was carried out using forward and reverse oligonucleotide primers (Operon, Germany) as given in Table 3. The numbers of cycles desired before reaching the plateau phase of amplification were determined for each gene. PCR amplification by Taq polymerase was conducted using a program of 94°C for 2 minutes, 55°C for 1 minute, and 72°C for 2 minute for *OsCam1-1*; *OsCam1-2*; *OsCam2*; and *OsCam3 *and a program of 94°C for 2 minutes, 58°C for 1 minute, and 72°C for 2 minute for *OsCam1-3*. PCR products were separated by agarose gel electrophoresis and visualized by ethidium bromide staining and UV fluorescencing. All enzymes and chemicals for RT-PCR were purchased from Promega (Madison, WI, USA).

## Authors' contributions

BB and TB participated in database searches and extensive analyses of the gene and protein sequences. BB carried out the laboratory work and prepared figures and tables. TB performed data analysis and interpretation, and drafted the manuscript. Both authors read and approved the final manuscript.

## Supplementary Material

Additional File 1 A list of all identified EF-hand-containing proteins as an Excel spreadsheet. Click here for file

Additional File 2 The alignment for the phylogenetic tree in Figure 1 as a ClustalX alignment file. Click here for file

Additional File 3 The alignment for the phylogenetic tree in Figure 2 as a ClustalX alignment file. Click here for file

Additional File 4 The alignment for the phylogenetic tree in Figure 7 as a ClustalX alignment file. Click here for file

## Figures and Tables

**Table 1:** Characteristics of *OsCam *and *OsCML *genes and the encoded proteins

**Name**	**Locus**^1^	**Chr**^2^	**cDNA length**^3^	**Amino Acids**^4^	**EF hands**^5^	**% of Met**^6^	**Identity to OsCaM1(%)**^7^	**Cys 27**^8^	**Lys 116**^9^	**Prenylation**^10^	**Myristoylation**^11^	**References**
*OsCam1-1*	LOC_Os03g20370	3	450	149	4	6.0	100.0	+	+			[10]
*OsCam1-2*	LOC_Os07g48780	7	450	149	4	6.0	100.0	+	+			
*OsCam1-3*	LOC_Os01g16240	1	450	149	4	6.0	100.0	+	+			
*OsCam2*	LOC_Os05g41210	5	450	149	4	6.0	98.7	+	+			[10]
*OsCam3*	LOC_Os01g17190	1	450	149	4	6.0	98.7	+	+			
*OsCML1*	LOC_Os01g59530	1	564	187	4	4.3	84.6			+		[8,9,10]
*OsCML2*	LOC_Os11g03980	11	552	183	4	4.9	70.3			+		
*OsCML3*	LOC_Os12g03816	12	552	183	4	4.9	68.9			+		
*OsCML4*	LOC_Os03g53200	3	465	154	4	6.5	68.9	+	+			
*OsCML5*	LOC_Os12g41110	12	501	166	4	4.8	62.2	+	+			
*OsCML6*	LOC_Os11g37550	11	513	170	4	6.5	53.9	+				
*OsCML7*	LOC_Os08g02420	8	447	148	2	2.8	47.7		+			
*OsCML8*	LOC_Os10g25010	10	576	191	4	5.2	47.0					
*OsCML9*	LOC_Os05g41200	5	468	155	1	3.2	46.1					
*OsCML10*	LOC_Os01g72100	1	558	185	4	4.3	45.6		+			
*OsCML11*	LOC_Os01g32120	1	636	211	4	1.4	44.1					
*OsCML12*	LOC_Os01g41990	1	750	249	4	2.8	43.9					
*OsCML13*	LOC_Os07g42660	7	510	169	4	5.3	43.6					
*OsCML14*	LOC_Os05g50180	5	522	173	4	4.6	43.3					
*OsCML15*	LOC_Os05g31620	5	606	201	4	4.0	40.7					
*OsCML16*	LOC_Os01g04330	1	546	181	4	3.9	40.5					
*OsCML17*	LOC_Os02g39380	2	495	164	4	4.9	37.7		+			
*OsCML18*	LOC_Os05g13580	5	477	158	4	5.7	37.7		+			
*OsCML19*	LOC_Os01g72550	1	441	146	3	7.5	37.2					
*OsCML20*	LOC_Os02g50060	2	525	174	4	4.0	35.3				+	
*OsCML21*	LOC_Os05g24780	5	594	197	3	4.6	35.3					
*OsCML22*	LOC_Os04g41540	4	753	250	4	3.6	35.2					
*OsCML23*	LOC_Os01g72540	1	456	151	3	7.9	35.1					
*OsCML24*	LOC_Os07g48340	7	594	197	3	3.0	33.9					
*OsCML25*	LOC_Os11g01390	11	450	149	3	6.7	33.6					
*OsCML26*	LOC_Os12g01400	12	450	149	3	6.7	33.6					
*OsCML27*	LOC_Os03g21380	3	573	190	3	3.2	33.3					
*OsCML28*	LOC_Os12g12730	12	519	172	4	4.8	33.1		+			
*OsCML29*	LOC_Os06g47640	6	513	170	3	4.1	33.1					
*OsCML30*	LOC_Os06g07560	6	711	236	4	2.1	32.8					
*OsCML31*	LOC_Os01g72530	1	456	151	3	5.3	31.6					
*OsCML32*	LOC_Os08g04890	8	591	196	3	2.6	30.2					

^1 ^The Institute of Genomics Research (TIGR) gene identifier number.

^2 ^Chromosome number in which the gene resides.

^3 ^Length of the coding region in base pairs.

^4 ^Number of amino acids of the deduced amino acid sequence.

^5 ^Number of EF hands based on the prediction by InterProScan.

^6 ^Percentage of methionine (M) residues in the deduced amino acid sequence.

^7 ^Number of identical residues divided by the total number of amino acids that have been aligned expressed in percentage.

^8 ^Presence of a cysteine equivalent to Cys26 of typical plant CaMs at residue 7(-Y) of the first EF-hand.

^9 ^Presence of a lysine equivalent to Lys115 of typical plant CaMs.

^10 ^Presence of a putative prenylation site.

^11 ^Presence of a putative myristoylation site.

**Table 2:** ESTs showing *OsCam *and *OsCML *expression in different tissues

***Cam/CML *name**	**Number of ESTs identified**							***Cam/CML *name**	**Number of ESTs identified**						
			
	**leaf**	**root**	**panicle**	**seed**	**callus**	**others**	**Total**		**leaf**	**root**	**panicle**	**seed**	**callus**	**others**	**Total**
*OsCam1-1*	106	17	49	6	14	89	281	*OsCML15*	5	1	8	-	3	37	54
*OsCam1-2*	34	13	17	7	11	38	120	*OsCML16*	20	8	12	2	1	43	86
*OsCam1-3*	21	5	9	-	9	15	59	*OsCML17*	1	-	10	-	-	6	17
*OsCam2*	35	7	25	5	17	36	125	*OsCML18*	11	2	88	-	1	17	119
*OsCam3*	57	5	30	6	23	29	150	*OsCML19*	-	-	-	-	-	-	0
*OsCML1*	30	-	4	1	2	6	43	*OsCML20*	-	-	7	-	-	2	9
*OsCML2*	-	-	-	-	-	1	1	*OsCML21*	-	2	-	-	-	-	2
*OsCML3*	61	-	26	6	6	22	121	*OsCML22*	4	-	35	-	2	9	50
*OsCML4*	20	5	2	2	2	7	38	*OsCML23*	-	-	-	-	-	-	0
*OsCML5*	-	-	-	-	1	2	3	*OsCML24*	10	4	1	-	-	16	31
*OsCML6*	-	-	-	-	-	-	0	*OsCML25*	-	-	-	-	-	-	0
*OsCML7*	18	-	6	4	3	33	64	*OsCML26*	1	-	1	-	-	1	3
*OsCML8*	13	1	4	-	1	4	23	*OsCML27*	21	2	7	3	5	30	68
*OsCML9*	1	-	-	-	-	3	4	*OsCML28*	-	-	1	-	-	2	3
*OsCML10*	16	7	19	-	1	30	73	*OsCML29*	6	2	1	-	-	4	13
*OsCML11*	7	-	1	-	2	-	10	*OsCML30*	10	13	7	2	2	21	55
*OsCML12*	3	1	1	-	-	1	6	*OsCML31*	11	5	5	1	5	19	46
*OsCML13*	13	4	109	-	4	12	142	*OsCML32*	-	-	2	-	-	2	4
*OsCML14*	-	-	-	-	2	10	12								

**Table 3:** Oligonucleotide primers used in this research

**Name**	**Sequence**
OsCaM1-1F	5'-GAAGCCAGGCTAAGCCCAGC-3'
OsCaM1-1R	5'-GCAAGCCTTAACAGATTCAC-3'
OsCaM1-2F	5'-CTTCGTTGATCCACTCACCC-3'
OsCaM1-2R	5'-ACACAATCTCCTCTGCCTTA-3'
OsCaM1-3F	5'-CCCCTCGCCGCCTCGCCACC-3'
OsCaM1-3R	5'-CCCATAACCAAATGCTGTCA-3'
OsCaM2-F	5'-GAGGAGGGTTCCCATTAAAT-3'
OsCaM2-R	5'-CGCAAGCTAAGCATCACAAT-3'
OsCaM3-F	5'-CCTTCCTCTCTCTCTCGCTC-3'
OsCaM3-R	5'-CCCCCTGTGTTGATCCAAAT-3'
